# Intimate Partner Violence in the COVID-19 Era: A Health, Psychological, Forensic and Legal Perspective

**DOI:** 10.3390/ijerph19094973

**Published:** 2022-04-20

**Authors:** Giussy Barbara, Alessia Viero, Irene Pellizzone, Laura Buggio, Federica Facchin, Cristina Cattaneo, Maria Elisa D’Amico, Paolo Vercellini, Alessandra Kustermann

**Affiliations:** 1 Gynaecological Unit and SVSeD, Service for Sexual and Domestic Violence, Fondazione IRCCS Ca’ Granda Ospedale Maggiore Policlinico, 20122 Milan, Italy; paolo.vercellini@policlinico.mi.it (P.V.); alessandra.kustermann@policlinico.mi.it (A.K.); 2 Department of Clinical Sciences and Community Health, University of Milan, 20122 Milan, Italy; 3 Legal Medicine and Toxicology Unit, Department of Cardio-Thoraco-Vascular Sciences and Public Health, University of Padua, 35121 Padua, Italy; alessia.viero85@gmail.com; 4 Department of Italian and Supranational Public Law, University of Milan, 20122 Milan, Italy; irene.pellizzone@unimi.it (I.P.); marilisa.damico@unimi.it (M.E.D.); 5 Department of Psychology, Catholic University of the Sacred Heart, Largo A. Gemelli 1, 20123 Milan, Italy; federica.facchin@unicatt.it; 6 Department of Biomedical Sciences for Health, University of Milan, 20133 Milan, Italy; cristina.cattaneo@unimi.it; 7 LABANOF—Laboratorio di Antropologia e Odontologia Forense, 20133 Milan, Italy

**Keywords:** intimate partner violence, domestic violence, gender violence, COVID-19, lockdown, family violence

## Abstract

This commentary aims to provide a multidisciplinary framework on intimate partner violence (IPV) during the COVID-19 pandemic (with a specific focus on the most predominant form of gender-based violence, i.e., male violence towards women), commenting on the multiple negative consequences of the pandemic on gender violence and providing elements of effective practice. We searched literature for reports/studies on the issue of IPV during the COVID-19 pandemic, focusing on health, psychological, forensic, and legal aspects. The combined effects of lockdowns, isolation at home with abusive partners, quarantine, and economic worries/loss of a job could significantly facilitate violence against women and, at the same time, diminish women’s chances to seek for help, with a strong negative impact on their life. The continued offer of clinical, psychological, forensic, and legal services for survivors of violence, despite the modifications to the provision of these services due to the new needs related to the COVID-19 pandemic, appears of utmost importance. All actions to support survivors of IPV are expected to be multidisciplinary, including the involvement of social and/or legal services and health systems, and woman-centred. Implementing these measures in the COVID-19 era appears challenging but is of primary importance.

## 1. Introduction

Intimate partner violence (IPV) represents one of the most common forms of violence against women, comprising any act of physical, sexual, psychological, and/or economic violence occurring in a family or in a couple relationship that presupposes cohabitation, with important negative consequences on physical and psychological health [1]. In addition, IPV also raises remarkable judicial, political, legal, sociological, and ethical issues.

Several reports exist on the rise of IPV during the COVID-19 pandemic [2,3,4,5], as the combined effects of lockdowns, isolation at home with abusive partners, quarantine, and economic worries/loss of a job could significantly facilitate violence against women and, at the same time, reduce women’s chances to seek help. For these reasons, the association between IPV and COVID-19 has quickly emerged as a shadow pandemic [3], raising awareness and worries worldwide.

From the beginning of the COVID-19 pandemic, there has been a significant intensification in women’s calls to the dedicated national helplines for domestic violence, compared with the same period in the previous year [6,7,8]. Specifically, in Italy (the first European nation that faced COVID-19), data from the Italian National Institute of Statistics [9] has reported that women’s calls to the national helpline against gender-based violence and stalking between 1 March and 16 April 2020 were 5031, with a significant increase (+75%) compared to the same period in 2019.

Despite an emerging literature on the association between COVID-19 and IPV, few studies have systematically examined the characteristics and types of women’s access to the dedicated IPV centres/shelters during the COVID-19 pandemic [4,10]. Available data [4,10] show a substantial reduction of the *in-person* requests for help to the dedicated services due to IPV. This observation seems to be in contrast with the increasing number of calls to the national helplines for domestic violence [4,10]. However, these data should be interpreted as an alarming signal of the severe limitation in women’s chances to seek help due to the pandemic-related restrictive measures (rather than a reduction of the episodes of gender violence), which deserves special attention.

Based on this background, we aim to provide a multidisciplinary framework on the issue of IPV during the COVID-19 pandemic (focusing on the most predominant form of gender-based violence (i.e., male violence towards women)), commenting on the multiple negative consequences of the pandemic on gender violence and discussing their practical implications. Specifically, we aim to focus on health, psychological, forensic, and legal dimensions related to IPV during the COVID-19 pandemic, also considering the social and health inequalities exacerbated by COVID-19 (and particularly for the most vulnerable people such as women exposed to IPV).

## 2. Social and Health Inequalities Heightened by COVID-19: A Major Hurdle for Vulnerable Women Exposed to IPV

IPV is deep-rooted in discrimination and structural inequality that is sustained by a complex range of social norms and expectations, policies, informal mechanisms, and gender stereotypes [11]. The COVID-19 pandemic has considerably exacerbated social, financial, and health-related inequalities [12,13], and concerns have emerged regarding women’s rights [11].

Previous experiences from past pandemics and/or natural calamities suggest that, among women, the physical and psychological burden significantly increases in times of emergencies [14]. With regard to the current COVID-19 pandemic, it has been reported that some governmental measures introduced to manage the spread of the infection and limit the impact of the pandemic on the national health systems had a tremendous negative impact on women’s sexual and reproductive health (for instance, in terms of limited access to safe abortion, contraception, reproductive health care, or quality maternal care) [11,15]. Difficulties in accessing women’s health facilities could be particularly harmful for women exposed to a constant coercive control by the abusive partner, in particular regarding reproductive aspects.

Furthermore, some COVID-19 related measures (such as lockdown, quarantine, home-working) might have had a detrimental impact on women’s economic independence [11], as women are more likely than men to be employed in less stable jobs [14]. A recent meta-analysis based on a total sample size of 44,772 women demonstrated that women’s economic empowerment is associated with a statistically significant decrease in IPV [16]. Therefore, the financial difficulties faced by women during the pandemic could increase economic violence within intimate relationships and therefore make it difficult for women suffering from IPV to leave their abusive partners and find a way out of violence [17].

Moreover, the decision to close schools in many countries to manage the COVID-19 pandemic might have been of particular concern for women, as women are more likely than men to look after their children or other family members who need continuous assistance, with a detrimental impact on their work and economic security [18]. In addition, especially in low-income countries, measures enacted to control the COVID-19 pandemic that preclude girls’ educational opportunities might make them more vulnerable to several forms of gender violence, such as child marriage and domestic violence [19].

## 3. Supporting IPV Survivors in Health Services during the COVID-19 Pandemic

About 30% of women worldwide have experienced some form of gender violence in their lifetime [20], with long-lasting adverse health consequences [21]. Several different diseases, particularly musculoskeletal, respiratory, cardiovascular and gastrointestinal disorders, and chronic pain diseases are more likely to be encountered in women who survived IPV [22]. Moreover, gynaecological disorders, including sexually transmitted infections, sexual dysfunctions, chronic pelvic pain, and adverse pregnancy outcomes, are prevalent in women suffering from domestic violence [23]. A possible explanation of this high frequency of chronic diseases could be attributed to high-stress levels and/or reduced healthy practice [24,25].

Thus, violence against women should be a public health priority. IPV is a worldwide prevalent phenomenon, affecting an extremely large number of women, with multiple negative repercussions on women’s health. Moreover, probably due to IPV-related long-lasting negative health consequences, women who have experienced gender violence are more likely to use health services than non-abused women [26]. In addition, IPV survivors may seek healthcare services before requesting help or support from anti-violence centres, social services and/or the police/judicial authorities. This is even more important in the context of the current COVID-19 pandemic, as dedicated supporting services or legal aid for women and their children may not have been (or even may not yet be) promptly available due to the restrictive policies enacted worldwide to contain the spread of the infection. For all of these reasons, the role of the health system in preventing violence against women in the COVID-19 era is vital.

At present, studies on the trends of requests for health care from IPV survivors during the COVID-19 pandemic are limited. However, some reports have indicated a rise in the access to health services due to IPV during the COVID-19 pandemic [27,28], whereas others found no differences in the number of trauma admissions related to episodes of domestic violence [29]. On the other hand, other authors have reported a substantial decrease in the number of women asking for *in-person* medical care and safety from violence during the COVID-19 pandemic at IPV dedicated services [4,10]. This reduction could be associated with the restrictive social measures, the increased likelihood of coercion by a violent partner at home, and to the fear of contracting the infection in health settings.

Health professionals have a unique opportunity to identify IPV victims, provide adequate counselling, and connect women to appropriate dedicated services, even when the violence is not spontaneously declared. However, during the COVID-19 pandemic, this opportunity has often been limited, as health services had to cancel and/or reschedule non-urgent clinical examinations, and/or since health operators were overwhelmed with the management of the COVID-19 cases and their ability to investigate other situations (such as the exposure to IPV) may have been limited [30].

Several authors have suggested different strategies to overcome the problem of offering health care to IPV survivors during the pandemic, including mobile health and telemedicine as effective techniques to support and counsel women [30,31,32,33,34,35,36,37,38,39].

Unquestionably, telemedicine should be supported as it could represent a primary instrument to meet women’s health requests. However, its limitations should be acknowledged. First, telemedicine cannot be appropriate for some health activities requiring *in-person* clinical evaluation, such as diagnostic techniques for imaging, description and photography of physical lesions due to violence, or swabbing for biological evidence. Second, technology-facilitated health services might be unsuitable for some women, as the abusive partner surveillance of phones, social media, and the internet may limit the capability of women exposed to IPV to ask for professional help [8], or even put the woman at risk of violence. Third, some women may not be familiar with online technology, and so unable to adequately use it [40].

The difficulties IPV survivors face during the COVID-19 pandemic must make health workers even more aware of the great importance of their role in recognizing, identifying, and supporting women experiencing domestic violence. In addition, educating key health professionals and/or providing a comprehensive and multidisciplinary service [41], including clinical, psychological and forensic care, integrated into existing health services (rather than as a stand-alone service, as also suggested by the WHO before the spread of the COVID-19 infection) [42], appear even more important in the context of limited accessibility to IPV services due to the pandemic.

## 4. IPV during the COVID-19 Pandemic: The Forensic Perspective

The collection of forensic evidence of violence in cases of IPV is essential from a legal perspective. During the COVID-19 pandemic, modifications to the current practice in the health care systems were made to prevent the pandemic from becoming an obstacle for forensic samples collection, which cannot be carried out telematically [43] as has happened for other aspects of the IVP ascertainment, such as preliminary evaluation, history collection, and eventual planning of all of the interventions [35,36,39,44,45,46,47].

In particular, in case of suspected or confirmed COVID-19 positive women reporting IPV, forensic medical services, starting from few guidelines published by forensic representative organisations [48,49,50], adjusted procedures to allow the proper collection of the forensic evidence, achieving a balance between the preservation of the integrity of forensic specimens, the prevention of viral contamination of samples, and the maintenance of practitioner safety [10,51].

Despite the risk of an emotionally traumatic experience in an unfamiliar and potentially dehumanizing environment, in examining women exposed to IPV forensic medical doctors started to dress in full PPE (personal protective equipment, consisting of gown, N95 mask, goggles/face shield, gloves), to minimize their COVID-19 exposure and that of the patients. Furthermore, a complete forensic sampling equipment that has to observe the DNA contamination minimization principles started to be also prepared in a “cold zone” (i.e., COVID-19 free zone) different from the “hot zone” (i.e., the zone where the IPV survivor affected by SARS-CoV-2 is placed) used to examine the patient. The collection of forensic samples and the procedure of packaging, sealing and labelling of the specimens has been adjusted to enable them to be safely collected and removed from the “hot zone”.

One of the main aspects of managing a forensic medical examination in a “hot zone” is the task of removing in a safe manner biological material and other evidentiary items (such as for example cloths) from the COVID-19 contaminated area. Usually the forensic material (i.e., a cardboard box containing forensic samples) is provided after the collection to attending police officers. During the COVID-19 pandemic, adjustments to usual packaging procedures became essential to prevent the forensic samples themselves unintentionally becoming fomites of SARS-CoV-2 transmission, putting examining practitioners/forensic medical doctors and police officers at risk for infection.

Finally, all COVID-19 related divergences from routine forensic procedures must be accurately documented to guarantee that forensic evidence achieved in a COVID-19 area is held to conventional high-quality standards to guarantee full admissibility in the criminal justice system.

Then there is the more complex issue of the clinical examination: in fact, the role of forensics is of paramount importance not only in collecting physical evidence but also in the entire procedure of diagnosing violence—type of trauma, timing, accidental versus non-accidental—all exquisitely medico legal issues, which require imaging and other types of hands-on technology. Operators tend to minimise the importance of such forensic clinical procedures with respect to other clinical disciplines as they tend not to appear as “important” or “lifesaving”, yet they are. Therefore, the recommendations are that a proper clinical forensic examination of the victim be performed with all of the measures taken for other medical acts, without excessively penalising or deferring it.

## 5. Psychological Issues Related to the Association between COVID-19 and IPV

Examining the association between COVID-19 and IPV from a psychological perspective is essential to identify pandemic-related factors that may increase the phenomenon. First, it has been suggested that the containment measures imposed by governments to limit the spread of the virus may represent a precipitating factor [4,52]. It is well known that isolating the woman is an important component of IPV [53]. In this regard, mandatory lockdowns exacerbated the isolation of women experiencing IPV and facilitated the abusers’ strategy, which is based on power and control [4]. Consequently, women were trapped at home with their abusive partner, with limited or absent social support, along with remarkable difficulties in seeking help [53,54]. As it has been recently reminded by Goodman and Epstein [53], isolation (which is an objective condition characterized by poor interactions with others) may be associated with loneliness (which is a subjective feeling, e.g., “I am alone”, “I have no one”) among IPV survivors, with dangerous consequences on their physical and mental health. In this regard, we agree with the authors on the importance of considering the psychological implications of stay-at-home and social distancing policies during the pandemic, especially in the context of IPV.

Besides isolation, the overall psychological impact of COVID-19 may have contributed to the increase in violent behaviours by exacerbating pre-existent mental disorders among perpetrators, including substance abuse [4,34].

The pandemic also led to remarkable changes in people’s lives and relationships, with a huge amount of stress among those who have been hit hard by the economic crisis. Women have been bearing the brunt of this situation: of 101,000 newly unemployed registered in Italy in December 2020, 99,000 were women [55]. These data are particularly alarming if one considers that economic dependence on the partner is another important risk factor for IPV [56]. Given these premises, identifying high-risk situations and developing new strategies to help women (for instance, using technology) is essential to save their lives.

## 6. COVID-19, Cohabitation with the Violent Partner and Economic Crisis: The Way Forward from a Legal Perspective

Psychological and physical health, and even the life of women and their children, are constantly endangered by their continuous and forced cohabitation with a violent man [57]. While this is true at any time, the pandemic emphasized the dramatic isolation of women and children exposed to IPV [58].

In legal terms, the pandemic raised awareness of the fact that home may be a dangerous place for women and children when IPV occurs. Therefore, the national and local authorities narrowed the “stay at home” policy in case of IPV: new solutions have to be explored since new women cannot be easily admitted to shelters due to the virus spreading-risk; judges have to look at the order of removal of the violent man from the family home as a suitable measure, since women cannot be immediately admitted in shelters due to the risk of spreading the virus; the suspension of all of the judicial proceedings and the judicial terms cannot operate in cases related to protection of essential rights in the family: the right to access justice for women is not impaired by the pandemics.

However, the real challenge in legal terms has to be assessed in the medium/long period. The national and regional lawmakers have to face the social and economic crisis stemming from the pandemic on women. Due to the still existing gender inequality, the earning of employed women is lower as compared to men and the gender pay gap persists: this is why we have to be aware that, due to the lack of economic independence, women’s path out of violence can be more challenging [59]. Consequently, the ability of public institutions and anti-violence centres to adopt measures and best practices for reintegrating women into work is fundamental in the medium and long term. These are necessary actions to enable women who find themselves in situations of double vulnerability to escape from the discomfort and humiliation of IPV, as indeed the Istanbul Convention (i.e., the Council of Europe Convention on preventing and combating violence against women and domestic violence) sets down, enhancing integrated policies [60].

## 7. Conclusions

The COVID-19 pandemic has highlighted new problems and challenges in preventing and combating the phenomenon of domestic violence.

The maintenance of clinical, psychological, forensic, and legal services for survivors of violence, despite the modifications to the provision of health, social and legal services due to the new needs due to the COVID-19 pandemic, appears of utmost importance [51,61,62,63,64].

Preventive measures to reduce violence against women, such as challenging discriminatory gender norms, reforming discriminatory family laws, reducing exposure to violence in childhood, supporting women’s access to employment and education, providing economic and social empowerment, are described as effective by the WHO’s RESPECT framework [65]. All actions mentioned above are expected to be multidisciplinary, including the involvement of social and/or legal services and health systems, and woman-centred. Implementing these measures in the COVID-19 era appears challenging but is primary importance.

Emergency times are characterized by the need to manage, with limited resources, new and enhanced needs of people, including the overall increase in violence, especially domestic violence, and the exacerbation of socioeconomic and health inequalities among vulnerable people, such as IPV survivors.

Raising the awareness of health professionals working in emergency rooms on gender violence appears to essential in supporting women who experienced IPV, especially in times of emergencies. Moreover, governments should include services for IPV survivors in COVID-19 emergency preparedness and response plans, and appropriately support and finance them.

In conclusion, the COVID-19 pandemic has turned the spotlight on IPV. The current pandemic is indeed a major emergency, but the parallel pandemic due to IPV represents an equally alarming situation, that needs to be extensively and systematically addressed. Health and legal systems should be considered as essential tools for preventing and combating IPV, but this should not merely rely on adopting ad hoc legislation, since integrated social and health policies are very much needed.
